# Negative feedback for *DARS2*–Fis complex by ATP–DnaA supports the cell cycle-coordinated regulation for chromosome replication

**DOI:** 10.1093/nar/gkab1171

**Published:** 2021-12-06

**Authors:** Kenya Miyoshi, Yuka Tatsumoto, Shogo Ozaki, Tsutomu Katayama

**Affiliations:** Department of Molecular Biology, Kyushu University Graduate School of Pharmaceutical Sciences, Fukuoka 812-8582, Japan; Department of Molecular Biology, Kyushu University Graduate School of Pharmaceutical Sciences, Fukuoka 812-8582, Japan; Department of Molecular Biology, Kyushu University Graduate School of Pharmaceutical Sciences, Fukuoka 812-8582, Japan; Department of Molecular Biology, Kyushu University Graduate School of Pharmaceutical Sciences, Fukuoka 812-8582, Japan

## Abstract

In *Escherichia coli*, the replication initiator DnaA oscillates between an ATP- and an ADP-bound state in a cell cycle-dependent manner, supporting regulation for chromosome replication. ATP–DnaA cooperatively assembles on the replication origin using clusters of low-affinity DnaA-binding sites. After initiation, DnaA-bound ATP is hydrolyzed, producing initiation-inactive ADP–DnaA. For the next round of initiation, ADP–DnaA binds to the chromosomal locus *DARS2*, which promotes the release of ADP, yielding the apo-DnaA to regain the initiation activity through ATP binding. This DnaA reactivation by *DARS2* depends on site-specific binding of IHF (integration host factor) and Fis proteins and IHF binding to *DARS2* occurs specifically during pre-initiation. Here, we reveal that Fis binds to an essential region in *DARS2* specifically during pre-initiation. Further analyses demonstrate that ATP–DnaA, but not ADP–DnaA, oligomerizes on a cluster of low-affinity DnaA-binding sites overlapping the Fis-binding region, which competitively inhibits Fis binding and hence the *DARS2* activity. DiaA (DnaA initiator-associating protein) stimulating ATP–DnaA assembly enhances the dissociation of Fis. These observations lead to a negative feedback model where the activity of *DARS2* is repressed around the time of initiation by the elevated ATP–DnaA level and is stimulated following initiation when the ATP–DnaA level is reduced.

## INTRODUCTION

**Figure 1.**
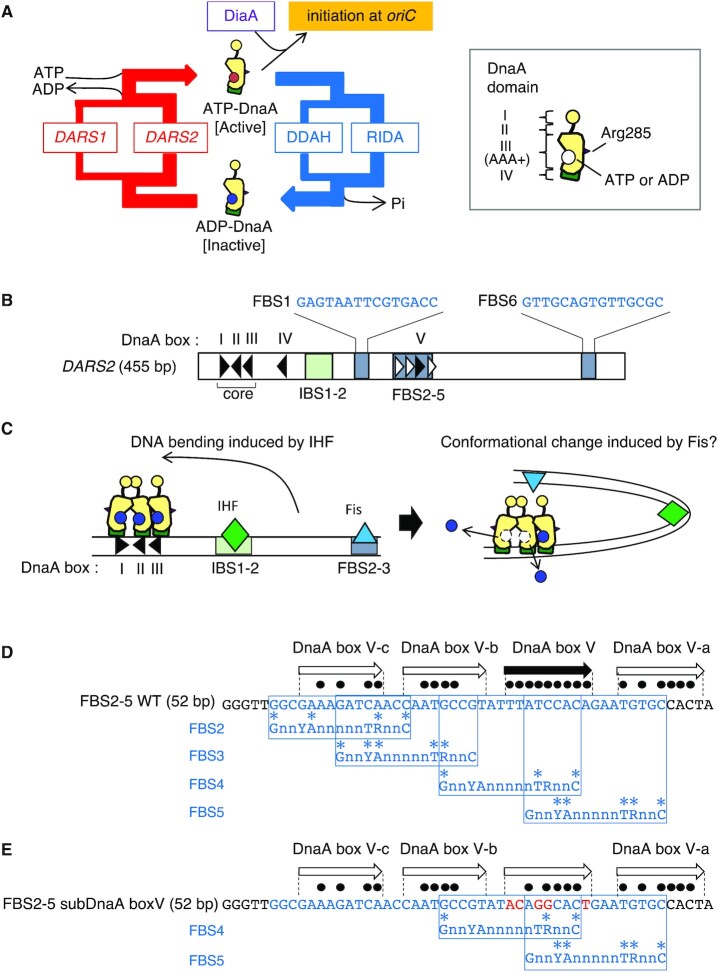
Cell cycle-coordinated DnaA activity and the structure of *DARS2*. (**A**) Schematic of the regulatory cycle (left) and domain structure (right) of DnaA. DnaA consists of four functional domains, of which the ATP/ADP-binding site resides in domain III (AAA+ domain). The arginine finger motif (Arg285) is indicated by a purple triangle. ATP–DnaA initiates replication by oligomerizing on *oriC*, stimulated by DiaA. After initiation, ATP–DnaA is converted to ADP–DnaA by two systems termed RIDA and DDAH, which promote DnaA–ATP hydrolysis in a manner dependent on the DNA-loaded clamp–Hda complex and the IHF-bound *datA*, respectively. *DARS1* and *DARS2*, which are genomic DNA elements, promote the generation of ATP–DnaA from ADP–DnaA by nucleotide exchange. *DARS2* is predominant over *DARS1 in vivo*, and is activated by the binding of IHF and Fis (see text for details). (**B**) The structure of *DARS2*. The 455-bp *DARS2* sequence is shown schematically. IHF-binding sites and Fis-binding sites are indicated by pale green and blue-gray boxes, respectively. The FBS1 and FBS6 DNA sequences are also shown. DnaA boxes and their orientation are indicated with triangles. Black triangles indicate typical DnaA boxes with the consensus sequence TT[A/T]TNCACA or the only one mismatch sequence (10). Low-affinity DnaA boxes identified in this study are indicated with an open triangle. IHF-binding consensus is TAAnnnnTTGATW (where W is A or T) (42). (**C**) Mechanistic model for DnaA–ADP dissociation from *DARS2*. ADP–DnaA, IHF and Fis bind to core DnaA boxes, IBS1–2 and FBS2–3, respectively. IHF bends the DNA, which could induce a conformational change in ADP–DnaA complex via interaction with Fis. As a result, ADP dissociates from multiple DnaA molecules, which are bound to DnaA boxes I and II and physically interact in a head-to-head manner by AAA+ domains (48). The sequence of the FBS2–5 fragment (**D**) and the mutant subDnaA box V (**E**). Black circles indicate bases matching the DnaA box consensus (TT[A/T]TNCACA). The Fis consensus binding sequence is GnnYAnnnnnTRnnC (Y = C or T; R = A or G) (51). Red letters represent introduced mutations.

In *Escherichia coli*, ATP–DnaA is required for the construction of a replication initiation complex at the chromosomal replication origin, *oriC* (1–4). This complex consists mainly of the DNA-bending protein IHF (integration host factor) and two DnaA pentamers held together by ATP-dependent DnaA–DnaA interactions facilitated by DnaA-binding sequences within *oriC*, called DnaA boxes (2–8). Recent studies have indicated that *oriC* contains two clusters of DnaA boxes (3,5,6,8,9). Notably, each cluster consists of a high-affinity DnaA box-consensus sequence (TT[A/T]TNCACA) neighboring four low-affinity, degenerated DnaA box sequences (3,5,6,10–12). DnaA tightly binds both ATP and ADP; however, only ATP promotes cooperative binding at *oriC* and the formation of the initiation complex, which promotes localized unwinding of the DNA duplex and successive loading of DnaB helicase onto the resulting single-stranded DNA (4,5,13–17). Upon replication initiation, DnaA binds to the high-affinity DnaA boxes individually, while ATP–DnaA cooperatively binds to low-affinity sites, resulting in the construction of two DnaA pentamers. DnaA-binding protein DiaA (DnaA initiator-associating protein), which exists as a homotetramer, simultaneously binds multiple DnaA molecules, stimulating cooperative binding of ATP–DnaA at *oriC* and replication initiation (18–21) (Figure 1A).

DnaA consists of four domains (2,4,22) (Figure 1A): domain I is required for the interaction with proteins such as DnaB and DiaA (16,19,20,22); domain II is a flexible linker (16,23); domain III contains ATPase-specific AAA+ motifs, including sites for ATP/ADP binding/hydrolysis and ATP-dependent DnaA–DnaA interactions, in addition to specific motifs for binding single-stranded DNA and an Arg finger (Arg285), which interacts with ATP bound to flanking DnaA molecules, promoting head-to-tail complex formation of ATP–DnaA molecules on *oriC* in a cooperative manner (11,24–29); and domain IV contains an HTH motif for specific binding to DnaA boxes (30).

Initiation of chromosomal replication is strictly regulated to occur only once per cell cycle. In *E. coli*, cellular levels of ATP–DnaA fluctuate during the cell cycle, peaking at replication initiation (31). Following initiation, DnaA-bound ATP is hydrolyzed by two systems: regulatory inactivation of DnaA (RIDA) and *datA*-dependent DnaA–ATP hydrolysis (DDAH) (1,2) (Figure 1A). RIDA is activated by the loading of DNA polymerase III holoenzyme onto DNA (32). The DNA-bound clamp subunit of this holoenzyme binds the AAA+ protein Hda, and the resulting complex interacts with ATP–DnaA molecules, promoting ATP hydrolysis and yielding inactive ADP–DnaA (32–35). This timely inactivation of DnaA is required for both repressing overreplication of the chromosome and cell viability (31–33,35–37). In addition, ADP–DnaA is degraded during stringent response (38). DDAH is required to sustain strict regulation of initiation by assisting RIDA (39,40). The *datA* locus is proximal to *oriC* and bears a cluster of DnaA boxes and IHF-binding site (IBS) (41,42). IHF binds to the *datA* IBS temporarily in the post-initiation stage, promoting ATP–DnaA complex construction and ATP hydrolysis at the *datA* locus (40,43) (Figure 1A).

To maintain accurate timing of replication initiation during the cell cycle in growing cells, reactivation of DnaA must be strictly regulated (1,2,44). The DnaA-reactivating sequence (DARS) system plays a central role in the timely increase of cellular ATP–DnaA, which is essential for coordinated replication initiation (45–49) (Figure 1A). The DARS system relies on two chromosomal loci, *DARS1* and *DARS2*, which share highly conserved core regions containing three uniquely arranged DnaA boxes (45) (I–III; Figure 1A and B). DnaA boxes I and II have opposing orientations, assisting the formation of dynamic homodimers of ADP–DnaA mediated by unique head-to-head interaction of the DnaA AAA+ domain (48). This interaction stimulates the dissociation of ADP, and the resultant apo-DnaA is released from *DARS* into solution, where it binds ATP molecules (45,48). DiaA stimulates ADP–DnaA assembly in the core region (48). In addition to this core domain, *DARS1* contains minimal stimulatory accessary regions, while *DARS2* contains further regulatory regions that allow for its specific activation during pre-initiation (46) (Figure 1B). This is consistent with the fact that *DARS2* plays a predominant role in the DnaA reactivation (45).

The *DARS2* regulatory region contains DnaA boxes, IHF-binding sites (IBS1–2) and multiple Fis-binding sites (FBS1–6, of which FBS2–5 form a cluster) (46) (Figure 1B). Unlike *DARS1*, *DARS2* is activated for the DnaA nucleotide exchange dependent on binding of both IHF and Fis (46). DNA bending by IHF could promote conformational changes of ADP–DnaA complexes via interaction with the bound Fis (Figure 1C). Fis is an abundant nucleoid protein that binds to specific DNA sequences as a homodimer, and is involved in a wide range of activities associated with DNA dynamics, including transcription, recombination, replication and construction of genome-wide tertiary structures (50,51). The cellular concentration of Fis fluctuates dramatically during growth phase, peaking in early exponential phase and disappearing in stationary phase (50,52). Thus, Fis is considered to be a crucial global regulator, directing cell proliferation. *DARS2* activation requires both the binding of IHF to IBS1–2 and the binding of Fis to FBS2–3 (46) (Figure 1B and C). We have previously shown that IHF binds specifically to IBS1–2 during pre-initiation and is released from *DARS2* during replication initiation (46). However, it is unclear whether the interaction between Fis and FBS2–3 is regulated in a cell cycle-coordinated manner. Moreover, regulatory mechanisms guiding the association and dissociation of these nucleoid proteins from *DARS2* are yet to be elucidated.

In this study, we reveal that Fis binds to *DARS2* FBS2–5 specifically during pre-initiation. Moreover, we show that *DARS2* is regulated through competitive binding of ATP–DnaA and Fis to FBS2–5. This FBS cluster contains DnaA box V and we identify novel, low-affinity DnaA boxes flanking box V, which together promote cooperative binding of ATP–DnaA. ATP–DnaA oligomerization competitively dissociates Fis from FBS2–3, leading to the inactivation of *DARS2*, coinciding with the cell cycle-coordinated oscillation of the Fis–FBS2–5 interaction *in vivo*. Taken together, we propose that DnaA regulates *DARS2* via a negative feedback loop, which leads to timely events during the chromosome replication cycle. In other words, the activity of *DARS2* is repressed around the time of initiation when the ATP–DnaA level is high and it is stimulated following initiation when the ATP–DnaA level is reduced by RIDA and DDAH. In this way, *DARS2* is proposed to play a critical role in the oscillation of the ATP–DnaA level over the cell cycle.

## MATERIALS AND METHODS

### Bacterial strains, plasmids, DNA fragments and media

The *E. coli* strains used in this study are listed in Supplementary Table S1. The λ-RED system and transduction using P1 phage were employed to introduce chromosomal mutations (53). Bacterial strains were grown in LB medium; M9 medium supplemented with 0.2% casamino acids, 5 μg/ml thiamine and 0.2% glucose; or Tryptone medium consisting of 10 g/l BD Bacto™ Tryptone and 10 g/l NaCl. Where necessary, antibiotics, arabinose (0.8%) and IPTG (1.0 mM) were included as indicated.

Plasmid pOA61tet is a pACYC177 derivative with wild-type *DARS2* adjacent to the *tetR* marker (46,47). pKX45tet and pMX21tet are derivatives of pOA61tet, which carry the subDnaA box V and subFBS6 alleles, respectively. To generate these plasmids, a 5.0-kb DNA fragment was amplified by PCR using pOA61tet and primers described in Supplementary Table S2 (Ksh-32/Ksh-33 for pKX45tet; MK-46/MK-47 for pMX21tet). PCR products were inserted into the plasmid vector by self-ligation. To generate pMX16tet, a pKX45tet derivative containing subFBS1 and subDnaA box V alleles, the same PCR and self-ligation methods were performed using pKX45tet and primers Ksh-3/Ksh-4 (Supplementary Table S2). To generate pTrcihfBA, which is an IHF overexpression plasmid with the *trc* promoter, a 1.3-kb DNA fragment carrying one copy of *ihfB* and two copies of *ihfA* was amplified using pOZihfBA3-1 and primers MK-90/MK-91 (Supplementary Table S2), digested PstI and XhoI, and ligated to PstI–XhoI fragments of pTrcHisC.

For the construction of mutant strains, 1.9-kb DNA fragments carrying a *DARS2* mutation and the *tet* gene were amplified from *DARS2* mutant plasmids (pKX45tet for subDnaA box V-*tet*, pMX21tet for subFBS6-*tet* and pMX16tet for subFBS1 subDnaA box V-*tet*) with primers D2TET-1 and mutH-2Nosite (Supplementary Table S2), as previously described (46). Fragments were transformed into MG1655 cells bearing pKD46 by electroporation, yielding strains TSU1, MYS29 and MYS18, respectively. *DARS2* WT-*tet* derived from MIT187, *DARS2* subFBS1-*tet* derived from KX5, or *DARS2* subFBS1 subDnaA box V-*tet* derived from MYS18 was introduced into KYA018 (*dnaC2*) using P1 transduction, yielding the strains MYS9, MYS1, and MYS19, respectively.

DNA fragments used for the EMSA were prepared by annealing the pairs of oligonucleotides listed in Supplementary Table S2A.

### Chromatin immunoprecipitation-coupled quantitative PCR

This assay was performed using synchronized *dnaC2* mutant cells as previously described (40,46). For cell cycle synchronization, cells were grown in M9 medium (15 ml) supplemented with 0.2% glucose and 0.2% casamino acids at 30°C until the *A*_660_ of the cultures reached 0.03. The temperature was then increased to 38°C for 90 min, before being shifted back down to 30°C. Samples were withdrawn at the times indicated, incubated in the presence of 3% formaldehyde to cross-link protein–DNA interactions and quenched with 125 mM of glycine, washing twice to remove residual formaldehyde.

For chromatin immunoprecipitation (ChIP), cells were lysed using lysozyme, sonicated to shear the DNA and ultracentrifuged to obtain a clear lysate. Five microliters was mixed with 1% SDS buffer to act as an input control. The remainder of the sample (350 μl) was incubated with polyclonal rabbit anti-Fis antiserum and Protein A Sepharose 4 Fast Flow at 4°C for 30 min with gentle rotation. Material bound to the Sepharose beads was recovered and dissolved in 1% SDS buffer. Input and ChIP samples were purified using Wizard SV Gel and a PCR Clean-Up Kit (Promega). Quantitative PCR (qPCR) was performed using SYBR Premix Ex Taq II and Thermal Cycler Dice TP800 (TaKaRa). Locus-specific primers used are described in Supplementary Table 2B and elsewhere (40,46) (ORI_1 and KW oriCRev for *oriC*, IHF-D2F and IHF-D2B for *DARS2*, RTYLCC-L and RTYLCC-R for *ylcC*, and TER_2 and SUEterRev1 for *ter*).

### Flow cytometry

Flow cytometry was performed as previously described (5,46). Briefly, cells were grown at 30, 37 or 42°C for >10 generations. At *A*_600_ of 0.1–0.2, 300 μg/ml rifampicin and 10 μg/ml cephalexin were added to the culture, which were incubated for a further 4 h to allow run-out replication. Subsequently, cells were fixed, stained with SYTOX Green and analyzed using FACS Calibur flow cytometry (BD Biosciences). In indicated cases, portions of growing cells were incubated in the presence of arabinose or IPTG for five generations before addition of rifampicin and cephalexin.

### Electrophoretic mobility shift assay

Electrophoretic mobility shift assay (EMSA) was performed as described previously (5). Briefly, DnaA was preincubated with 3 μM ATP or ADP, before further incubation with or without Fis at 30°C for 5 min in 5 μl buffer G [20 mM HEPES–KOH (pH 7.6), 80 mM potassium chloride, 1 mM EDTA, 4 mM DTT, 5 mM magnesium acetate, 0.1 mg/ml bovine serum albumin, and 10% (v/v) glycerol] containing 35 nM of 52-bp DNA and competitor λ-DNA (25 or 50 ng as indicated). DNA–protein complexes were separated on a 7% polyacrylamide gel by electrophoresis at 120 V for 120 min in Tris–borate buffer and stained with GelStar.

### Pulldown assay

A biotinylated 70-bp DNA fragment bearing wild-type or mutant *DARS2* (37.5 or 15 nM) was preincubated on ice for 10 min in buffer G (40 μl) containing Fis at the concentrations stated. DNA–protein complexes were recovered using streptavidin-coated beads (Promega), resuspended in buffer G (40 μl) containing 400 nM FBS1 DNA as a Fis-binding competitor and incubated with ATP–DnaA or ADP–DnaA in the presence or absence of DiaA, followed by pulldown experiments. When DnaA was preincubated similarly with DNA in buffer G containing FBS1, Fis was added for further incubation, followed by pulldown experiments. Samples were washed in 40 μl buffer G′ (buffer G devoid of bovine serum albumin), and the material was retained on the beads resuspended in 10 μl SDS sample buffer. Samples were analyzed by SDS-PAGE on a 15% polyacrylamide gel followed by silver staining.

## RESULTS

### The cell cycle-coordinated oscillation of Fis–*DARS2* binding

Previously, we revealed by the ChIP-coupled qPCR (ChIP-qPCR) method that IHF binds to *DARS2* IBS1–2 in a cell cycle-coordinated manner (46). In contrast, no specific changes in Fis binding to *DARS2* were detected. However, in the present study, we considered that Fis binding to FBS2–3 may be regulated in a manner distinctive from other FBS. FBS2–3 sites are essential for the activation of *DARS2* and the others are not (46) (Figure 1B and C).

**Figure 2.**
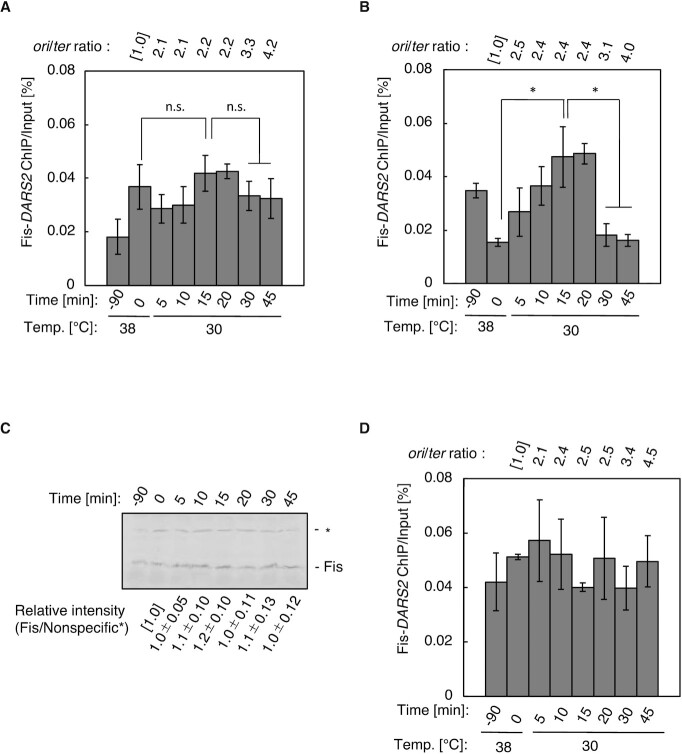
The Fis–*DARS2* interaction oscillates in a cell cycle-coordinated manner. Fis-ChIP analysis following cell cycle synchronization was performed in MYS9 cells (*dnaC2 DARS2* WT) (**A**) or isogenic mutant strains MYS1 (*dnaC2 DARS2* subFBS1) (**B**, **C**) and MYS19 (*dnaC2 DARS2* subFBS1 subDnaA box V) (**D**). The *ylcC* region without Fis-binding sequences was used as a background control. Error bars represent SD from at least five independent experiments. *P < 0.05; n.s., not significant (two-tailed Student’s *t*-test). To monitor synchronization, the copy numbers of *oriC* and *terC* were deduced using qPCR and the ratios (ori/ter) were shown as relative to the value at time 0. Expression levels of Fis in MYS1 were deduced by western blotting using anti-Fis antibodies (**C**). A protein cross-reacting with anti-Fis antibody, which is indicated by the asterisk, was used as a loading control. Fis protein levels relative to time 0 were shown.

The nonessential FBS1 is located close to FBS2–5, which may interfere with specific analysis of Fis binding to FBS2–5 in our previous ChIP-qPCR analysis. Therefore, we first analyzed Fis binding in FBS1-substituted mutants. FBS1 was replaced with DNA sequences with no specific affinity for Fis (*subFBS1*) in a *dnaC2* (Ts) genetic background: a temperature-sensitive *dnaC* mutant background that allows for temperature-dependent cell cycle synchronization (40,46). In these cells, initiation at *oriC*, but not ongoing replication, is severely inhibited at high temperatures (54). Cells were incubated for 90 min at 38°C before the temperature was shifted to 30°C, allowing a first round of replication initiation within 5 min of the temperature shift and a second round of replication 30–45 min later (39,40,46,54–56). ChIP-qPCR was performed, showing that there were no specific changes in Fis–*DARS2* binding in *dnaC2* cells, as shown previously (46) (Figure 2A). An exceptional difference from the previous data was seen only at the time immediately after temperature shift down, which could be caused by changing of Fis binding to FBS1 and subtle differences in experimental conditions (see later). In addition, the ratios of *oriC/ter* supported the occurrence of the first and second rounds of initiation in a timely manner (Figure 2A). Notably, *dnaC2 subFBS1* cells showed oscillation in the binding of Fis to *DARS2* similar to IHF (46) (Figure 2B). The cellular level of Fis was constant in *dnaC2 subFBS1* cells throughout the experiment (Figure 2C), suggesting that oscillation in Fis binding was unrelated to Fis protein level. Further ChIP-qPCR experiments using nonsynchronized cultures identified that Fis binding to *DARS2* was markedly reduced in FBS2–3-substituted mutant cells compared with wild-type cells, while substitution of FBS6 had little effect (Supplementary Figure S1). Like FBS1, FBS6 is nonessential for *DARS2* activation (46). These data suggest that FBS6 distant from FBS2–5 resides substantially outside of detectable regions by this ChIP-qPCR analysis. Together, it is suggested that Fis binds to *DARS2* FBS2–5 prior to the initiation of replication and is released upon initiation.

Next, we investigated the importance of the DnaA box V flanking FBS2–3 in the regulation of Fis binding (Figures 1B and D, and 2D). DnaA box V was replaced with the DNA sequence that retained the Fis-binding consensus sequence but disrupted DnaA binding (Figure 1D and E). In these cells, Fis–*DARS2* binding did not oscillate but remained relatively constant, indicating a crucial role for the DnaA box V sequence in the regulation of Fis dissociation from FBS2–5 (Figure 2D).

### Negative regulation of replication initiation requires *DARS2* DnaA box V

**Figure 3.**
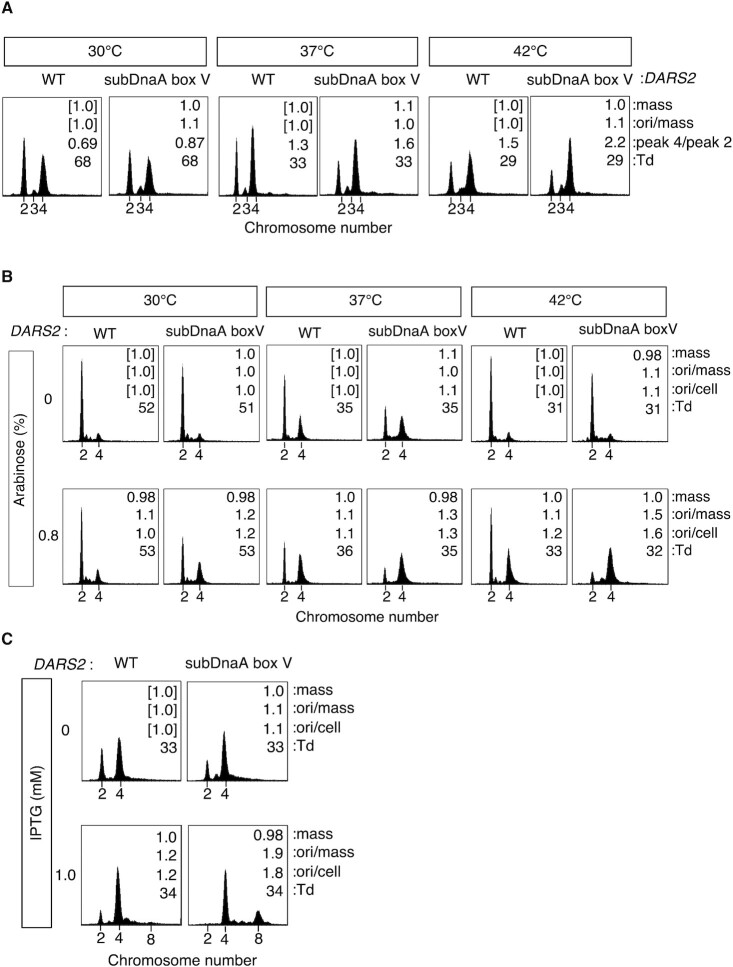
*DARS2* DnaA box V plays a negative role in replication initiation. Analysis of flow cytometry experiments performed with MIT187 (WT) and TSU1 (subDnaA box V) cells growing in supplemented M9/glucose/CAA medium (**A**) or with those cells with pOZihfBA3-1 growing in Tryptone medium in the presence or absence of 0.8% arabinose (**B**) at each temperature. Also, similar analysis was performed using those cells with pTrcihfBA growing in supplemented Tryptone/0.2% glucose medium in the presence or absence of 1.0 mM IPTG at 37°C (**C**). Mean values of cell mass, ori/mass, ori/cell and doubling time of cells (*T*_d_) were determined from three biological replicates and are shown relative to WT cells grown in the absence of arabinose or ITPG (WT standards are shown as [1.0]). The ratios of cells with four copies of the *E. coli* chromosome relative to cells with two copies were calculated (peak 4/peak 2).

Next, we investigated the impact of *DARS2* DnaA box V on the regulation of replication initiation. Cells were grown to exponential phase and further incubated in the presence of rifampicin and cephalexin, which inhibit replication initiation (but not elongation) and cell division, resulting in run-out replication. Analyzing cell size and DNA content by flow cytometry allowed us to deduce the copy number of replication origins and examine replication initiation events (5,43,46). MG1655 cells and an isogenic *DARS2* DnaA box V mutant (subDnaA box V) were grown at 30, 37 or 42°C in M9/glucose/CAA medium, before flow cytometry analysis was performed (Figure 3A). The wild-type and mutant cells grew with similar doubling times. The majority of cells contained either two or four chromosomes, with fewer wild-type cells displaying the four-chromosome phenotype compared with subDnaA box V cells. This phenotypic difference was clearer at 42°C than at 30°C. Strict regulation of *DARS2* would be more crucial in cells grown at higher temperatures for rapid growth. These data suggest that *DARS2* DnaA box V plays a role in repressing replication initiation, basically consistent with previous data (45).

*DARS2* activation is dependent on both IHF and Fis. Even in the subDnaA box V mutants, timely binding and dissociation of IHF to and from *DARS2* should be preserved, restricting the *DARS2* activation level for DnaA. Therefore, we next examined the effect of IHF overexpression on replication. MG1655 and subDnaA box V cells were transformed with pOZihfBA3-1, a derivative of pBAD18 carrying the IHF coding genes *ihfA* and *ihfB*, downstream of an arabinose-inducible promoter (7), and the resulting strains were analyzed for cell size and DNA content as mentioned earlier. When IHF expression was induced in the wild-type *DARS2* cells at 42°C, the four-chromosome peak was moderately increased compared with cells without induction (Figure 3B, 42°C), consistent with previous data (57). However, in subDnaA box V cells grown under the same conditions, IHF overexpression dramatically stimulated replication initiation, diminishing the two-chromosome peak while increasing the four-chromosome peak. When cells were grown at 30 or 37°C (Figure 3B), initiation was only mildly stimulated by IHF overexpression, a phenotype further enhanced by mutation of DnaA box V (Figure 3B). Next, we infer that clearer overinitiation phenotype could appear in medium including glucose, which accelerates cell growth. As the arabinose-inducible promoter is repressed by glucose, we replaced it with the *trc* promoter and performed similar experiments using medium including glucose. When IHF expression was induced by IPTG, replication initiation was evidently stimulated even at 37°C depending on subDnaA box V (Figure 3C). These results suggest that increased IHF and Fis binding at the *DARS2* locus stimulated by IHF overexpression and a lack of DnaA binding to DnaA box V, results in overactivation of DnaA and increased replication initiation.

### ATP–DnaA oligomerization at *DARS2* FBS2–5 requires DnaA box V

**Figure 4.**
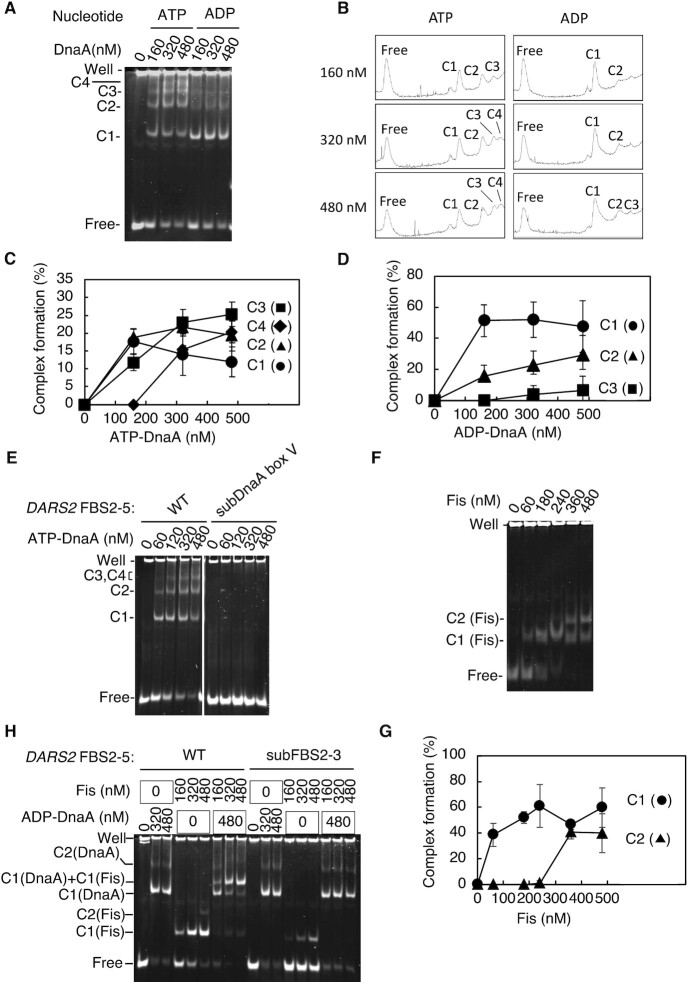
DnaA box V facilitates formation of ATP–DnaA oligomers on *DARS2*. (**A**–**D**) ATP–DnaA-specific oligomerization. EMSA with increasing concentrations of ATP–DnaA or ADP–DnaA protein and 35 nM FBS2–5 WT DNA (175 fmol). Representative images from four independent experiments are shown (**A**). Free, protein-free DNA; C1–C4, complexes 1–4. Lanes were analyzed by densitometry using ImageJ, and the profiles for 160, 320 and 480 nM DnaA are shown (**B**). Band intensities corresponding to protein-free DNA and complexes C1–C4 were quantified using ImageJ (**C**, **D**). Graphs show mean intensities with error bars representing SD from at least four independent experiments. SEM at each data point was <10%. (**E**) DnaA box V mutant analysis. The indicated amounts of ATP–DnaA were coincubated with FBS2–5 WT or its derivative bearing FBS2–5 subDnaA box V, followed by EMSA. (**F**, **G**) Fis binding to FBS2–5. The indicated amounts of Fis were coincubated with FBS2–5 WT, followed by EMSA. A representative gel image is shown (**F**). Band intensities for protein-free DNA (Free) and Fis-bound complexes, C1 (Fis) and C2 (Fis), were quantified and plotted as a percentage of total DNA (**G**). Error bars represent SD from at least two independent experiments. (**H**) Independent binding of ADP–DnaA and Fis to FBS2–5. ADP–DnaA (0–480 nM) was incubated with FBS2–5 or FBS mutant (subFBS2–3) in the presence or absence of Fis (0–480 nM), followed by EMSA.

We have shown earlier that Fis temporarily binds to *DARS2* FBS2–5 in a pre-initiation stage (Figure 2B). As FBS2–5 contains a DnaA box, we considered whether this release may result from cooperative binding and oligomerization of ATP–DnaA at this region. Cooperative binding of ATP–DnaA to *oriC* during the construction of the initiation complex has been shown previously. ATP–DnaA interacts with a flanking ATP–DnaA molecule via the AAA+ Arg finger motif, stimulating ATP–DnaA oligomerization on a cluster of low-affinity DnaA boxes (5,11,29). Consistently, we found that FBS2–5 consists of DnaA box V flanked by repeats of low-homology DnaA box-consensus sequences (DnaA boxes V-a, V-b and V-c in Figure 1D). These probable low-affinity DnaA boxes are aligned at 2–3 bp intervals, which has been shown to most efficiently facilitate cooperative DnaA–DnaA binding in a head-to-tail manner (8). We therefore analyzed DnaA binding to this region by EMSA, using purified proteins. DnaA efficiently constructed oligomers on wild-type FBS2–5 fragments in the presence of ATP but not ADP, with trimers (C3) constituting one of the major proportions of oligomers when ATP–DnaA concentrations were ≥480 nM (Figure 4A–D and Supplementary Figure S2). Higher ATP–DnaA concentrations slightly yielded FBS2–5 with four DnaA molecules (C4). ADP–DnaA bound to FBS2–5 largely as a monomer (Figure 4A–D). Similar results were shown for ATP–DnaA R285A, a mutant of the Arg finger that supports cooperative binding (Supplementary Figure S2B). For determination of the bound DnaA molecules, we referred EMSA data of an *oriC* fragment (R5MI2, 55 bp) bearing four low-affinity DnaA boxes, R5M, τ2, I1 and I2. Various previous analyses support that ATP–DnaA tetramers are largely constructed on the *oriC* fragment (5,6,8,11,12,58). When a similar *oriC* fragment (R5MI2 R1_4_) with high-affinity sequences of DnaA box R1 replacing the four low-affinity boxes was used, ADP–DnaA yielded monomer to trimer complexes at suboptimal DnaA concentrations (Supplementary Figure S2A–C) (5). Migration positions of DnaA complexes C1–C4 constructed on FBS2–5 (52 bp) were similar to those of the *oriC* fragments (Supplementary Figure S2B and C).

Notably, disruption of *DARS2* DnaA box V inhibited ATP–DnaA binding completely, suggesting that ATP–DnaA binds first to DnaA box V before oligomerization (Figure 4E). Moreover, individual substitution of DnaA box V-a or V-b, but not V-c, with sequence defective for DnaA binding considerably inhibited trimer formation on FBS2–5, while disruption of all the low-affinity DnaA boxes diminished ATP–DnaA oligomerization completely, without inhibiting ATP–DnaA binding to DnaA box V (Supplementary Figure S3). Interestingly, DnaA box V-a substitution considerably inhibited the formation of all DnaA oligomers. ADP–DnaA was able to bind to FBS2–5 *in vitro*; however, concurrent binding of multiple DnaA molecules was severely inhibited (Figure 4A–E), consistent with data obtained for DnaA binding at *oriC*. Together, these data suggest that ATP–DnaA initially binds to *DARS2* DnaA box V and, in the presence of ATP–DnaA, recruits further ATP–DnaA molecules to the low-affinity sites within FBS2–5. It should be noted that DnaA box V is located outside of FBS2–3, the essential sites for *DARS2* activation, while DnaA box V-b overlaps FBS2–3 (Figure 1C).

### ATP–DnaA competes with Fis to bind FBS2–5

Next, we compared the affinity of DnaA and Fis for FBS2–5 fragments. As previously reported, Fis bound to FBS2–5 predominantly as a monomer or dimer (46) (Figure 4F and G). Each binding site within FBS2–5 overlaps partly with neighboring sites (Figure 1C), which is likely to physically restrict Fis binding. Fis C1 and C2 complexes were formed at Fis concentrations of 100–200 and ≥360 nM, respectively (Figure 4F and G), largely comparable to the concentrations required for ATP–DnaA oligomerization (Figure 4A–E). This binding and dimerization were diminished when FBS2–3 was mutated (subFBS2–3; Figure 4H), suggesting Fis binds predominantly to this region critical for *DARS2* activation. Both ADP–DnaA and Fis bound to the FBS2–5 fragment independently. Fis binding to subFBS2–3 fragments, but not wild-type fragments, was hampered even in the presence of ADP–DnaA binding (Figure 4H). These data suggest that Fis predominantly binds FBS2–3 in the presence of ADP–DnaA, consistent with the finding that *DARS2* is activated *in vivo* when ADP–DnaA is abundant.

**Figure 5.**
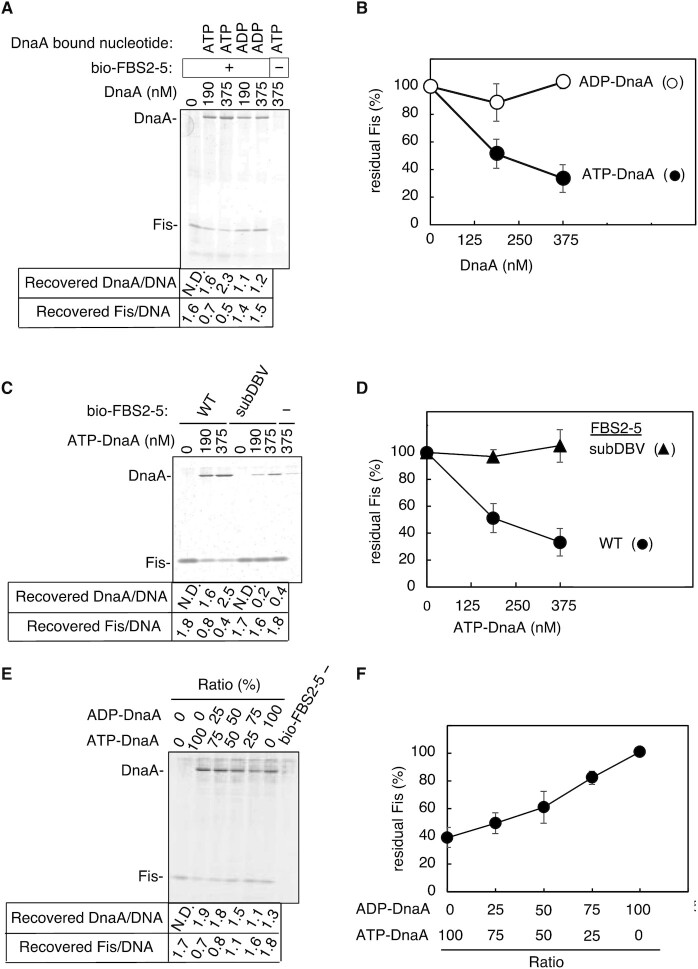
ATP–DnaA oligomers compete with Fis on *DARS2*. (**A**–**F**) Competitive pulldown assay. 5′-Biotinylated FBS2–5 (WT) DNA or a DnaA box mutant (subDnaA box V, subDBV) (37.5 nM) was preincubated with Fis (375 nM), and the Fis–DNA complexes were isolated, followed by further incubation with the indicated concentrations of ATP–DnaA or ADP–DnaA (**A**–**D**), or with mixtures of ATP–DnaA and ADP–DnaA at the indicated ratios (375 nM DnaA in total) (**E**, **F**). Recovered proteins were analyzed by SDS-PAGE and silver staining, followed by densitometry using ImageJ. Recovered DnaA and Fis relative to bio-FBS2–5 (DNA) are shown below representative gel images (**A**, **C**, **E**) and plotted as a percentage of Fis recovered in the absence of DnaA (**B**, **D**, **F**). Means and SD from three independent experiments are shown.

Next, we performed pulldown experiments to assess binding competition. Biotin-labeled *DARS2* FBS2–5 (bio-FBS2–5) was incubated with Fis, and Fis-bound FBS2–5 was isolated. ATP–DnaA or ADP–DnaA was added in increasing concentrations, and DNA binding was analyzed by bio-FBS2–5 pulldown experiments. ATP–DnaA, but not ADP–DnaA, effectively displaced Fis bound to FBS2–5 in a dose-dependent manner (Figure 5A and B). This means that ATP–DnaA can lead to Fis dissociation much better than ADP–DnaA, which is likely resulting from enhanced cooperative binding to FBS2–5 of ATP–DnaA rather than ADP–DnaA (see later). The number of recovered DnaA molecules per DNA was reasonable, considering that weakly bound DnaA molecules would be partly dissociated during washing in the final steps. Experiments performed with bio-FBS2–5 mutated for DnaA box V showed that both ATP–DnaA binding and Fis dissociation were dependent on DnaA box V (Figure 5C and D). In addition, similar experiments were performed using bio-FBS2–5 and mixtures of ATP–DnaA and ADP–DnaA at various ratios. Fis dissociation was shown according to the proportion of ATP–DnaA (Figure 5E and F). Together, these data suggest that binding of ATP–DnaA to FBS2–5 leads to the accumulation of DnaA oligomers, which competitively inhibit Fis binding.

**Figure 6.**
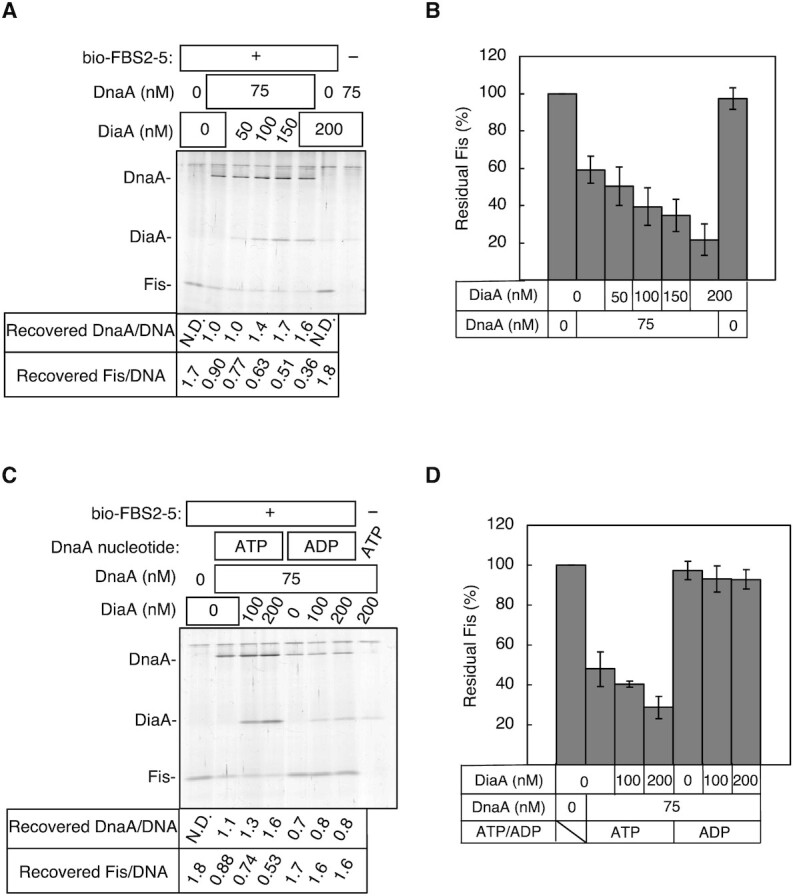
DiaA stimulated assembly of ATP–DnaA and displacement of Fis on FBS2–5. (**A**–**D**) Competitive pulldown assay in the presence of DiaA. bio-FBS2–5 (15 nM) was preincubated with Fis (200 nM) and the bio-FBS2–5–Fis complexes were isolated and incubated with ATP–DnaA or ADP–DnaA and DiaA at the concentrations shown. Recovered proteins were analyzed by SDS-PAGE and silver staining. Recovered DnaA and Fis relative to bio-FBS2–5 (DNA) are shown below representative gel images (**A**, **C**) and plotted as a percentage of Fis recovered in the absence of DnaA (**B**, **D**). Means and SD from at least four independent experiments are shown.

Finally, we analyzed the effect of DiaA on Fis displacement. DiaA forms a homotetramer with each protomer binding to a specific site on DnaA domain I, which stimulates the assembly of ATP–DnaA on *oriC* (19,20) and of ADP–DnaA on the *DARS1* core (48). When a suboptimal amount of DnaA was used to observe the effects of DiaA, bio-FBS2–5 pulldown experiments showed that Fis dissociation was stimulated by DiaA in the presence of ATP–DnaA, but not ADP–DnaA (Figure 6A–D). These data suggest that DiaA promotes ATP–DnaA loading onto *DARS2* FBS2–5, resulting in Fis dissociation.

### Fis binding to FBS2–5 in mixtures of ATP–DnaA and ADP–DnaA

**Figure 7.**
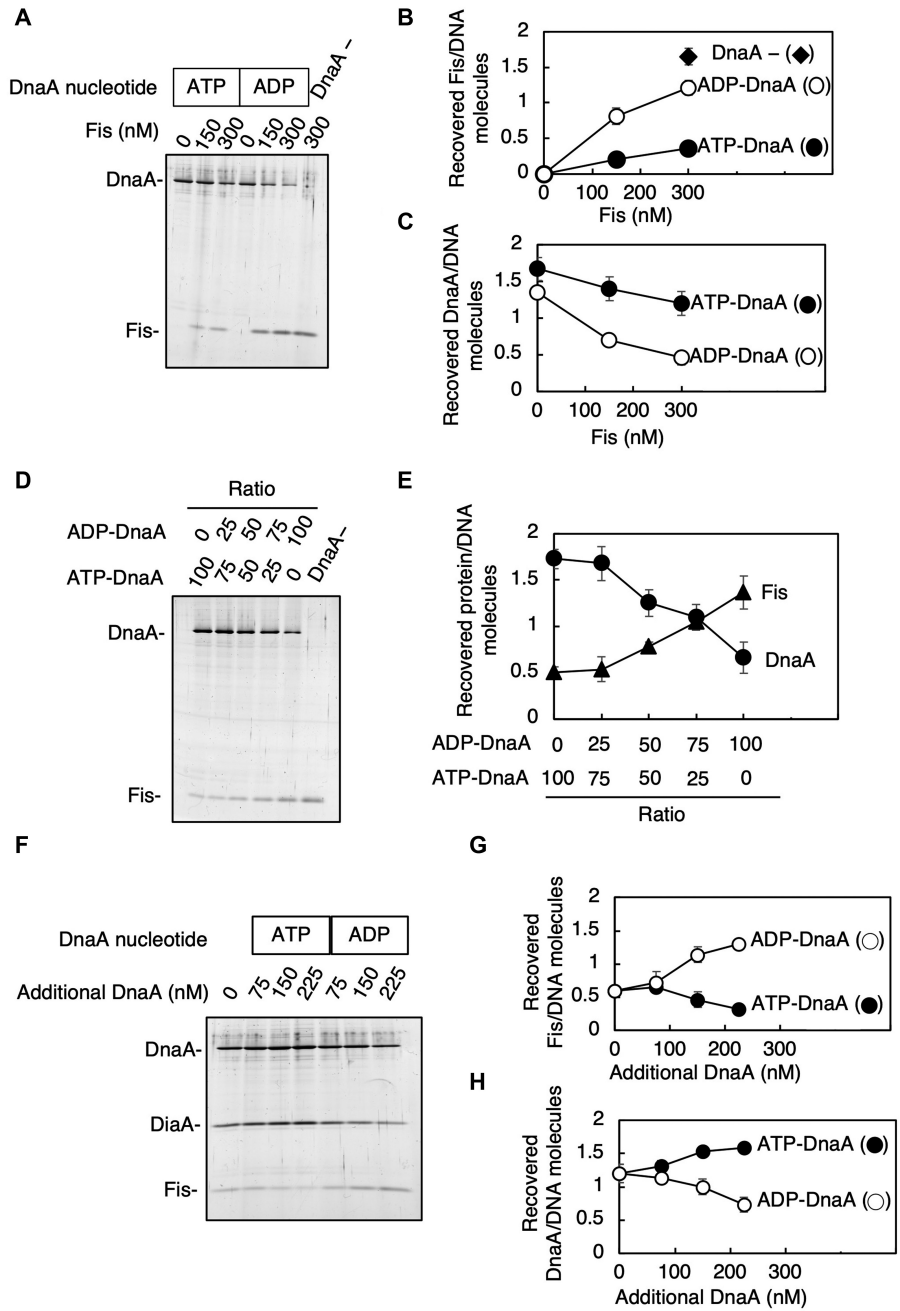
Fis binding to FBS2–5 was resumed with ADP–DnaA. (**A**–**C**) Competitive pulldown assay using FBS2–5 preincubated with ATP–DnaA and ADP–DnaA. Bio-FBS2–5 (37.5 nM) was preincubated on ice for 10 min with ATP–DnaA or ADP–DnaA (400 nM), followed by further incubation on ice for 10 min with the indicated concentrations of Fis. Recovered proteins were analyzed by SDS-PAGE and silver staining. Representative gel image (**A**) and the recovered molecules of DnaA and Fis relative to DNA are plotted (**B**, **C**). Means and SD from three independent experiments are shown. (**D**, **E**) Competitive pulldown assay using mixtures of ATP–DnaA and ADP–DnaA. Similar pulldown assay was performed using mixtures of ATP–DnaA and ADP–DnaA at the indicated ratios (400 nM in total). Preincubation of bio-FBS2–5 with the mixtures was followed by further incubation with Fis (300 nM). Representative gel image (**D**) and the plots of the recovered molecules of DnaA and Fis relative to DNA (**E**) are shown. Means and SD from three independent experiments are shown. (**F**–**H**) Competitive pulldown assay using FBS2–5 preincubated with ATP–DnaA in the presence of DiaA. Bio-FBS2–5 (15 nM) was preincubated with ATP–DnaA (75 nM) and DiaA (150 nM), followed by further incubation with Fis (200 nM) in the presence or absence of ATP–DnaA or ADP–DnaA at the indicated concentrations. Representative gel image (**F**) and the plots of the recovered molecules of DnaA or Fis relative to DNA (**G**, **H**) are shown. Means and SD from three independent experiments are shown.

We further performed pulldown experiments to assess Fis binding to FBS2–5 preincubated with DnaA. Bio-FBS2–5 was incubated with ATP–DnaA or ADP–DnaA, followed by further incubation with increasing amounts of Fis. In the absence of DnaA, Fis was recovered at similar efficiency to similar experiments shown in Figure 5 (Figure 7A and B). Preincubation with ATP–DnaA, but not ADP–DnaA, effectively inhibited Fis binding to FBS2–5 (Figure 7A and B). ATP–DnaA was only slightly dissociated in elevated amounts of Fis and ADP–DnaA binding was inhibited by Fis more effectively (Figure 7A and C). When mixtures including ATP–DnaA and ADP–DnaA at various ratios were preincubated, Fis binding was allowed in a manner dependent on ADP–DnaA level (Figure 7D and E). These results are consistent with the idea that ATP–DnaA oligomers constructed on FBS2–5 inhibit Fis binding, and ADP–DnaA binding to DnaA box V allows Fis binding to FBS2–3 (Figures 4 and 5). Next, ATP–DnaA and DiaA were preincubated with bio-FBS2–5, which was followed by further incubation with Fis in the presence or absence of ATP–DnaA and ADP–DnaA. Addition of ADP–DnaA, but not ATP–DnaA, increased Fis binding in a dose-dependent manner (Figure 7F–H). These data suggest that when ADP–DnaA levels are increased, cooperative binding of ATP–DnaA to FBS2–5 is inhibited, allowing Fis binding.

### Conservation of *DARS2* DnaA box V in γ-proteobacterial genomes

**Figure 8.**
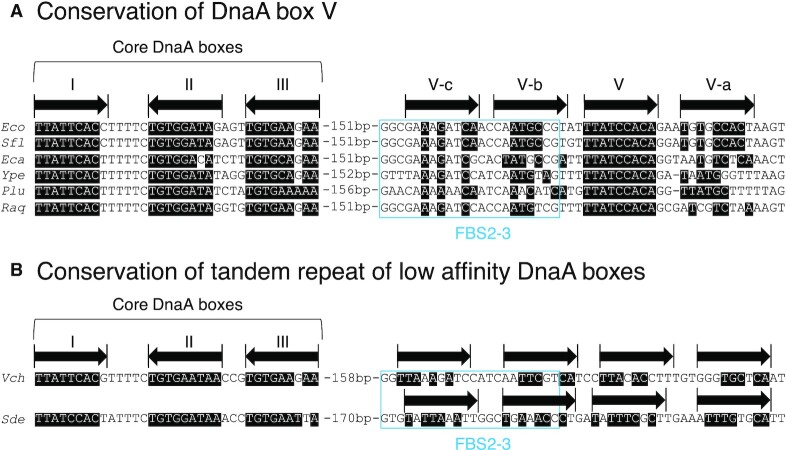
DnaA box sequences are conserved in *DARS2*-homologous sequences in γ-proteobacteria related to *E. coli*. Homology to *E. coli DARS2* core, FBS2–3 and DnaA boxes was analyzed across seven *E. coli*-proximal bacterial species. All sequences were taken from the NCBI database. Arrows indicate DnaA box orientation. Bases identical to the DnaA box-consensus sequence (TT[A/T]TNCACA) are indicated in white. FBS2–3 regions with Fis-binding consensus, defined in a previous study (46), are marked with a blue rectangle. Some species conserve full consensus DnaA box (DnaA box V) and proximal low-affinity DnaA boxes (**A**), and the others conserve only repeats of low-affinity DnaA boxes (**B**). Eco, *Escherichia coli* *K-12*; Sfl, *Shigella flexneri 2a 2457T*; Eca, *Erwinia carotovora atroseptica SCRI1043*; Ype, *Yersinia pestis KIM*; Plu, *Photorhabdus luminescens TTo1*; Raq, *Rahnella aquatilis*; Vch, *Vibrio cholerae El Tor N16961*; Sde, *Shewanella denitrificans OS217*.

Fis is conserved among γ-proteobacterial species related to *E. coli* (59). We previously reported that FBS2–3 sequences were also highly conserved in these species, including pathogens with conserved Fis homologs and *DARS2* core sequences (46). Upon examination of the *DARS2* sequence from representative γ-proteobacterial species, we discovered that a cluster of low-affinity DnaA box sequences with overlapping Fis-binding consensus was similarly conserved in addition to the DnaA box V sequence conserved in a part of the species (Figure 8): some species conserve both three low-affinity DnaA boxes and a complete consensus box corresponding to the DnaA box V (Figure 7A), and others conserve a tetrad of low-affinity DnaA boxes (Figure 8B). This suggests that in these species, which include pathogenic bacteria, the *DARS2* regulatory mechanism is conserved.

## DISCUSSION

Strict and dynamic regulation of cellular ATP–DnaA is essential for the temporal regulation of replication initiation. In this study, we showed that *DARS2*, an element essential for the conversion of ADP–DnaA into ATP–DnaA, is regulated in a cell cycle-dependent manner via a negative feedback loop. Fis, a crucial activator protein for *DARS2*, binds to *DARS2* FBS2–3, an essential site for *DARS2* activation, during pre-initiation stage, increasing cellular ATP–DnaA. During replication initiation, ATP–DnaA forms a trimer or tetramer on *DARS2* FBS2–5, dissociating Fis and preventing *DARS2* activation. This regulation is a simple yet elegant, fundamental system that efficiently regulates cellular ATP–DnaA and replication initiation. Timely repression of *DARS2* would be effective for maximizing substantial rates of DnaA–ATP hydrolysis after initiation, ensuring strict regulation (Figure 1A).

In addition, this study has demonstrated that Fis plays an important role in cell cycle regulation. Fis is depleted during stationary phase and has been recognized as a growth phase regulation factor widely involved in recombination, transcription and replication (50,52). While Fis interaction with *oriC* has been analyzed, mutant and biochemical analyses have indicated that direct interaction between Fis and minimal *oriC* is unimportant for the regulation of replication initiation during the cell cycle (60–65). However, Fis has been shown to regulate many other genomic sites, including virulence genes in pathogenic bacteria (66–69). A possibility of participation of ATP–DnaA in regulation of Fis binding to such sites cannot be excluded.

**Figure 9.**
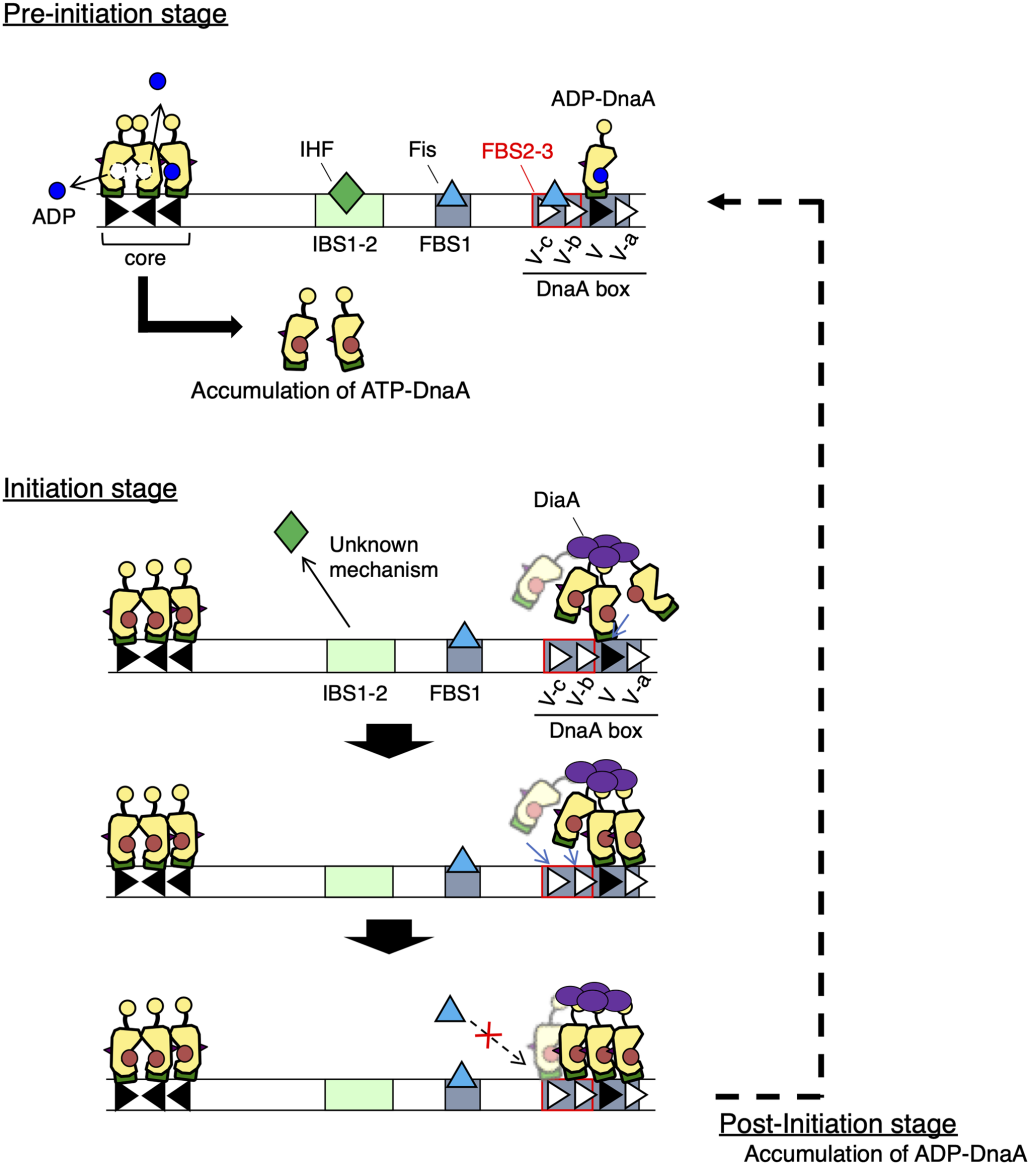
A model to describe cell cycle-dependent regulation of *DARS2* via a negative feedback loop. This model describes the activation and inactivation of *DARS2* via DnaA-mediated regulatory control of Fis binding. During pre-initiation, ADP–DnaA is abundant and occupies DnaA box V, allowing Fis to bind to FBS2–3. Binding of both ADP–DnaA and Fis promotes the formation of ADP–DnaA–IHF–Fis–*DARS2* complexes, which further promote DnaA nucleotide exchange and the accumulation of ATP–DnaA. During initiation, ATP–DnaA levels peak and ADP–DnaA is replaced with ATP–DnaA at DnaA box V by chemical equilibrium. This stimulates cooperative binding of further ATP–DnaA molecules facilitated by DiaA tetramers. The resulting ATP–DnaA–DiaA complexes span portions of FBS2–5, competitively restricting Fis access to the DNA, resulting in the inactivation of *DARS2*. Concurrently, IHF is released from *DARS2* by an as yet unknown mechanism. After initiation, DnaA–ATP hydrolysis is promoted, accumulating ADP–DnaA molecules, which inhibits cooperative binding of ATP–DnaA.

In this study, we propose a novel mechanism for the regulation of Fis binding by a cluster of DnaA boxes, consisting of a high-affinity DnaA box V and three low-affinity DnaA boxes, V-a, V-b and V-c (Figure 9). DnaA box V has complete sequence homology with the 9-mer DnaA box-consensus sequence, while V-a to V-c share four to six bases of homology (Figure 1D). While ADP–DnaA predominantly bound only to DnaA box V (Figure 4A, B and D), the loading of ATP–DnaA onto DnaA box V allowed cooperative ATP–DnaA binding to the low-affinity boxes (Figure 4E). During pre-initiation, ADP–DnaA is abundant and binds to DnaA box V located within FBS4–5, which physically obstructs Fis binding to FBS4–5. This in turn directs Fis to load onto DnaA box V-proximal FBS2–3, which is essential for *DARS2* activation (Figure 4H). The loading of Fis and ADP–DnaA leads to the formation of ADP–DnaA–IHF–Fis–*DARS2* complexes, which are capable of producing ATP–DnaA via nucleotide exchange (46) (Figure 1C). Thus, ATP–DnaA accumulates during pre-initiation, leading to the replacement of ADP–DnaA with ATP–DnaA on DnaA box V. As replication initiation approaches, ATP–DnaA levels peak and three or four molecules of ATP–DnaA cooperatively bind to FBS2–5, using DnaA box V-bound ATP–DnaA as an assembly core (Figure 9), leading to Fis dissociation. Based on the direction of DnaA boxes within FBS2–5, ATP–DnaA molecules form oligomers via head-to-tail interaction, which are similar to those formed in *oriC*–DnaA complexes (2,5,11). This assembly, and therefore Fis dissociation, is stimulated by DiaA (Figure 9).

DnaA box V-a is more important for the formation of ATP–DnaA oligomers than DnaA boxes V-b and V-c (Supplementary Figure S3). This suggests that ATP–DnaA binding to DnaA box V-a contributes to the stabilization of ATP–DnaA–DnaA box V complexes, stimulating the formation of higher multimers. DnaA box V-b partially overlaps FBS2–3 (Figure 1D), particularly with the Fis-binding consensus sequence within FBS3. ATP–DnaA binding to DnaA box V-b is likely to be essential for the inhibition of Fis binding, acting as a simple, physical obstacle. In addition, FBS1 could contribute to the titration of Fis molecules dissociated from FBS2–3, assisting in *DARS2* regulation, consistent with our previous observation that disruption of *DARS2* FBS1 provoked a slight increase in replication initiation compared with the wild-type *DARS2* sequence (46).

Nucleotide-exchange activities that occur at *DARS2* require simultaneous binding of IHF and Fis. The *DARS2*–IHF interaction also occurs during pre-initiation (46). Dissociation of either IHF or Fis is sufficient to inactivate *DARS2*; however, both IHF and Fis dissociate from *DARS2* together. The reason for this is unclear, but it is possible that multiple redundant regulatory pathways act as a fail-safe, as the regulation of *DARS2* function must be strict. Also, IHF and Fis bind many genomic loci and regulate many cellular functions. Genome-wide analyses have revealed that IHF- and Fis-binding sites exist in ∼3000 locations in the *E. coli* genome (70,71). Dissociation of IHF and Fis from *DARS2* may allow binding of these proteins to other sites. Finally, Fis and IHF binding to DNA influences the tertiary structure of the whole genome. Simultaneous binding and dissociation of IHF and Fis might be related to the modulation of this tertiary structure. Transposition of *DARS2* to a region proximal to *terC*, the replication terminus of the chromosome, partly inhibits replication initiation (47,49). Changes in the tertiary structure of this region may explain this observation. At present, IHF–*DARS2* binding/dissociation mechanisms are under investigation.

In the replication origin *oriC*, clusters of low-affinity DnaA-binding sites promote oligomer formation of ATP–DnaA molecules, playing a positive role for replication initiation (1–5). In *DARS2*, the similar mechanism has been revealed to play a negative role for replication initiation. This means that ATP–DnaA plays both positive and negative roles during the cell cycle as the bacterial cell cycle engine. Furthermore, this cell cycle-coordinated regulation by a cluster of low-affinity DnaA boxes could be widely conserved in other fundamental cellular processes, including transcription. Transcription of the *nrdAB* operon, which encodes a nucleotide reductase that regulates *de novo* deoxyribonucleotide synthesis, is regulated in a cell cycle-coordinated manner (72,73). The *nrdAB* promoter contains at least two DnaA boxes, and construction of ATP–DnaA oligomers within this region is thought to interfere with the loading of RNA polymerase, thus inhibiting transcription (74). Careful examination of the *E. coli* genome for low-affinity DnaA box clusters would be of great interest.

## DATA AVAILABILITY

The data supporting the findings of this study are available from the corresponding author upon reasonable request.

## Supplementary Material

gkab1171_Supplemental_File
