# Supplementary material for: Negative feedback for DARS2–Fis complex by ATP–DnaA supports the cell cycle-coordinated regulation for chromosome replication
Source: Nucleic Acids Res. 2021 Dec 6;49(22):12820–35. doi: 10.1093/nar/gkab1171 (PMC8682772; doi:10.1093/nar/gkab1171)
Supplement: gkab1171_Supplemental_File [file gkab1171_supplemental_file.pdf]

Supplementary Data

Negative feedback for *DARS2*-Fis complex by ATP-DnaA supports the cell cycle-coordinated regulation for chromosome replication

Kenya Miyoshi, Yuka Tatsumoto, Shogo Ozaki, and Tsutomu Katayama*

Department of Molecular Biology, Kyushu University Graduate School of Pharmaceutical Sciences, Fukuoka, Japan

Supplementary Table S1: List of *E. coli* strain

Strain	Relevant genotype	Reference or Source
MG1655	Wild type	Laboratory stock
MIT187	MG1655 *DARS2* WT-*tet*	Kasho *et al*., 2014
KYA018	MG1655 *dnaC2 zjj18*::*cat*	Kasho *et al*., 2014
MYS9	KYA018 *DARS2* WT-*tet*	This work
MYS1	KYA018 *DARS2*subFBS1-*tet*	This work
MYS18	MG1655 *DARS2*subFBS1 subDnaA box V-*tet*	This work
MYS19	KYA018 *DARS2*subFBS1 subDnaA box V-*tet*	This work
TSU1	MG1655 *DARS2*subDnaA boxV-*tet*	This work
KX54	MG1655 *DARS2*subFBS2-3-*tet*	Kasho *et al*., 2014
MYS29	MG1655 *DARS2*subFBS6-*tet*	This work

Reference

Kasho, K., Fujimitsu, K., Matoba, T., Oshima, T. & Katayama, T. Timely binding of IHF and Fis to *DARS2* regulates ATP-DnaA production and replication initiation. *Nucleic Acids Res*. 42, 13134–13149 (2014).

Supplementary Table S2: List of oligonucleotides

A. ssDNA used for production of dsDNA

ssDNA	Sequences	dsDNA Product
FBS2-5-f	GGGTTGGCGAAAGATCAACCAATGCCGTATTT ATCCACAGAATGTGCCACTA	FBS2-5 WT
FBS2-5-r	TAGTGGCACATTCTGTGGATAAATACGGCATT GGTTGATCTTTCGCCAACCC	
FBS2-5 subDnaA box V-f	GGGTTGGCGAAAGATCAACCAATGCCGTATAC AGGCACTGAATGTGCCACTA	FBS2-5 subDnaA box V
FBS2-5 subDnaA box V-r	TAGTGGCACATTCAGTGCCTGTATACGGCATT GGTTGATCTTTCGCCAACCC	
FBS2-5 subV-a-f	GGGTTGGCGAAAGATCAACCAATGCCGTATTT ATCCACAGAACGCGCCGCCA	FBS2-5 subDnaA box V-a
FBS2-5 subV-a-r	TGGCGGCGCGTTCTGTGGATAAATACGGCATT GGTTGATCTTTCGCCAACCC	
FBS2-5 subV-b-f	GGGTTGGCGAAAGATCAACCGGCGCCGCATTT ATCCACAGAATGTGCCACTA	FBS2-5 subDnaA box V-b
FBS2-5 subV-b-r	TAGTGGCACATTCTGTGGATAAATGCGGCGCC GGTTGATCTTTCGCCAACCC	
FBS2-5 subV-c-f	GGGTTGGCGGGGGGCCGACCAATGCCGTATTT ATCCACAGAATGTGCCACTA	FBS2-5 subDnaA box V-c
FBS2-5 subV-c-r	TAGTGGCACATTCTGTGGATAAATACGGCATT GGTCGGCCCCCCGCCAACCC	
FBS2-5 subV-abc-f	GGGTTGGCGGGGGGCCGACCGGCGCCGCATTT ATCCACAGAACGCGCCGCCA	FBS2-5 subDnaA box V-abc
FBS2-5 subV-abc-r	TGGCGGCGCGTTCTGTGGATAAATGCGGCGCC GGTCGGCCCCCCGCCAACCC	
R5-I2wt-f	TCATTAACTGTGAATGATCGGTGATCCTGGAC CGTATAAGCTGGGATCAGAATGA	*oriC* R5MI2 WT
R5-I2wt-r	TCATTCTGATCCCAGCTTATACGGTCCAGGAT CACCGATCATTCACAGTTAATGA	
R5-I2 4R1-f	TCATTACCTGTGGATAACCTGTGGATAACCTG TGGATAACCTGTGGATAAAATGA	*oriC* R5MI2 $R1_4$
R5-I2 4R1-r	TCATTTTATCCACAGGTTATCCACAGGTTATC CACAGGTTATCCACAGGTAATGA	

B. Primers used for construction of mutant strains or quantitative PCR

Primers	Sequences
D2TET-1	CACTAATAACAATTGAATAACTCACAGTTATGTGCAGAGTTATAAACAG AAATTCTCATGTTTGACAGC
MutH-2 Nosite	ATTGCTTTTTAGGTTGCCG
Ksh-3	ACATATGTTTTTCATTACTAAAATTACATGTAATGCATTG
Ksh-4	TCGATACGCAGGTCACACCTCTCATTTACGGG
Ksh-32	GACTGAATGTGCCACTAAGTTAAGCAC
Ksh-33	CATGTATACGGCATTGGTTGATCTTTC
MK-46	TGTTTCGGAACTCGATTACCGGCAACCTAAAAAGC
MK-47	CTGCAATGGGTTTTCCAAATCTGGTCACTG
ORI_1	CTGTGAATGATCGGTGATC
KW oriCRev	GTGGATAACTCTGTCAGGAAGCTTG
IHF-D2F	GTCACACCTCTCATTTACGGG
IHF-D2B	CCAGTTTTTAGTGGTTCAGTGC
RTYLCC-L	GGCGTGGTAAAGGGTATCG
RTYLCC-R	TCTGCGGGGTGATGGTAAAG
TER_2	TATCTTCCTGCTCAACGGTC
SUEterRev1	GAACTACGCGGGAAATACC
MK-90	AAGCCTCGAGCAACAGCAGCCGCTTAATTTG
MK-91	TGCATGCCTGCAGGTCGACTCTAG

Supplementary Figure S1. Fis-ChIP analysis with subFBS6

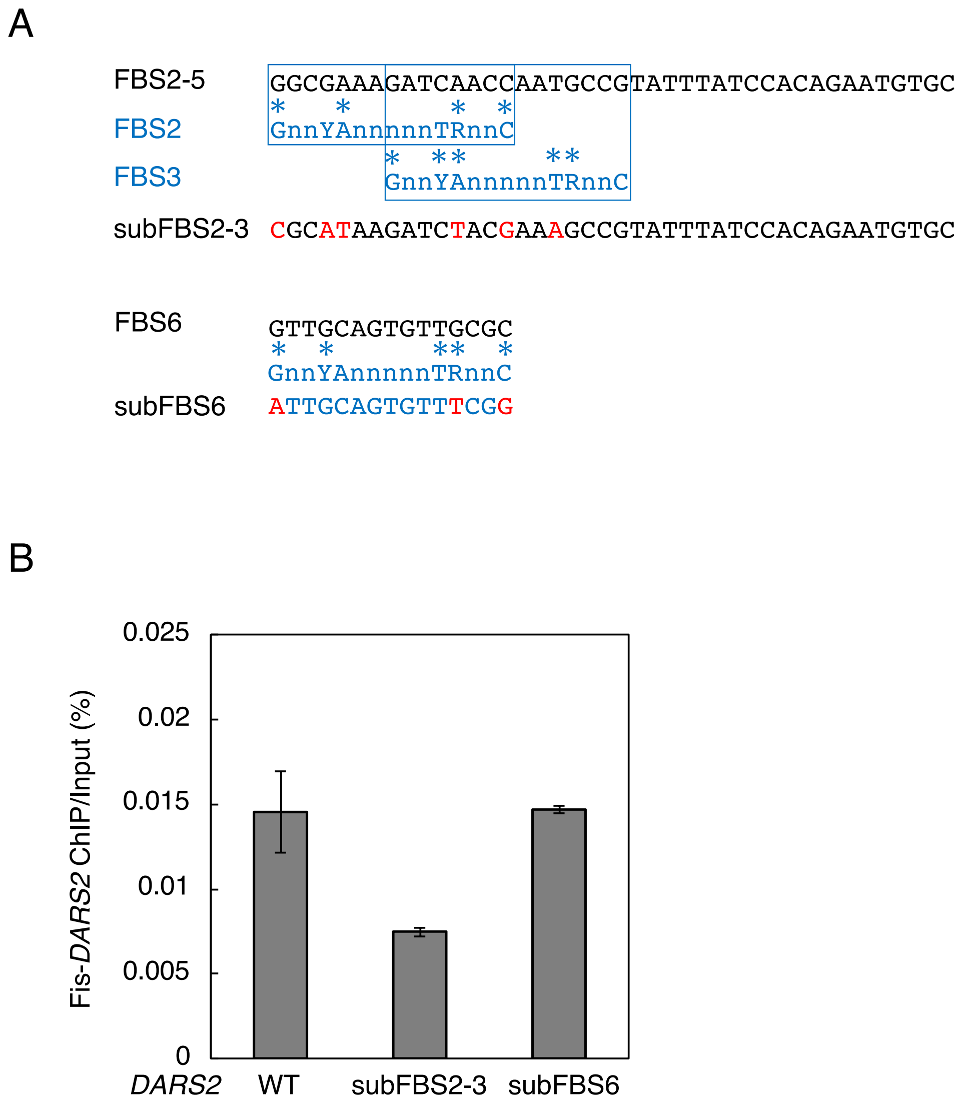

A. Schematic view of mutations introduced to FBS2-3 (top) and FBS6 (bottom). The native sequences which are identical to the FBS consensus are indicated by asterisks. Substituted bases are displayed by red letters.

B. Fis-ChIP analysis with subFBS2-3 or subFBS6 mutant cell. MIT187 (WT *DARS2*), KX54 (*DARS2* subFBS2-3), MYS29 (*DARS2* subFBS6) cells were grown at 30°C in supplemented M9/glucose/caa medium until the A_{660} reached 0.2. The Fis-*DARS2*/Input (%) was calculated as described in Figure 2. Error bars represent SD from three independent experiments.

Supplementary Figure S2. EMSA experiments using *oriC* fragments or DnaA muntant

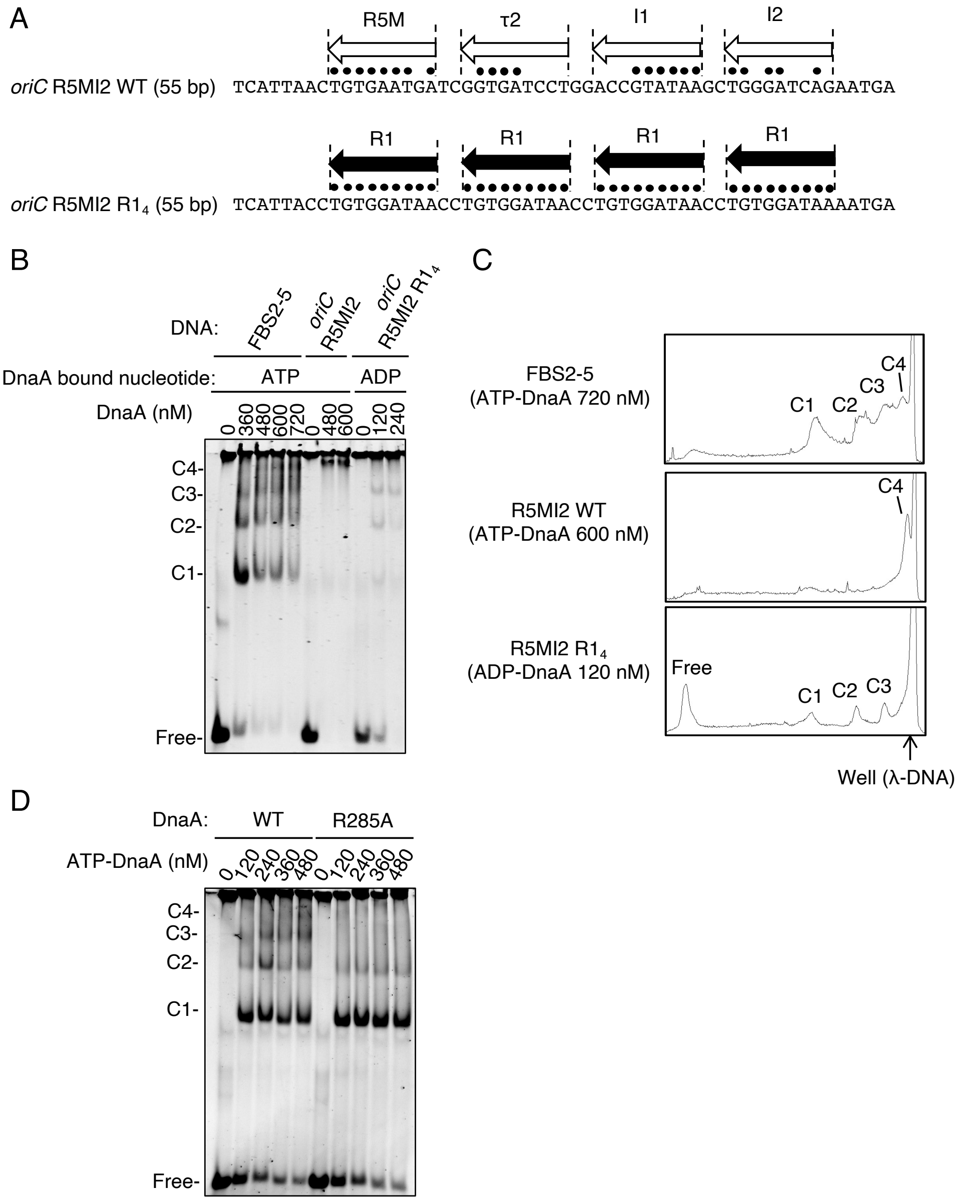

A. Sequences of *oriC* R5MI2 WT and R5MI2 $R1_4$ fragments. The minimal *oriC* includes a cluster of DnaA boxes R5M, τ2, I1 and I2. Symbols are the same as those used in Figure 1D. White and black arrows indicate low-affinity DnaA boxes and high-affinity DnaA boxes, respectively.

B-C. EMSA with the indicated amounts of ATP-DnaA or ADP-DnaA and 35 nM (175 fmol) FBS2-5 WT DNA, *oriC* R5MI2 WT, or *oriC* R5MI2 $R1_4$. Representative image is shown (**B**). Free, Protein-free DNA; C1-C4, Complex 1-4. Lanes were analyzed by densitometry using Image J, and the profiles are shown (**C**).

D. EMSA experiments with the indicated amounts of ATP-DnaA WT or R285A and 35 nM (175 fmol) FBS2-5 WT DNA.

Supplementary Figure S3. Determination of ATP-DnaA binding sites

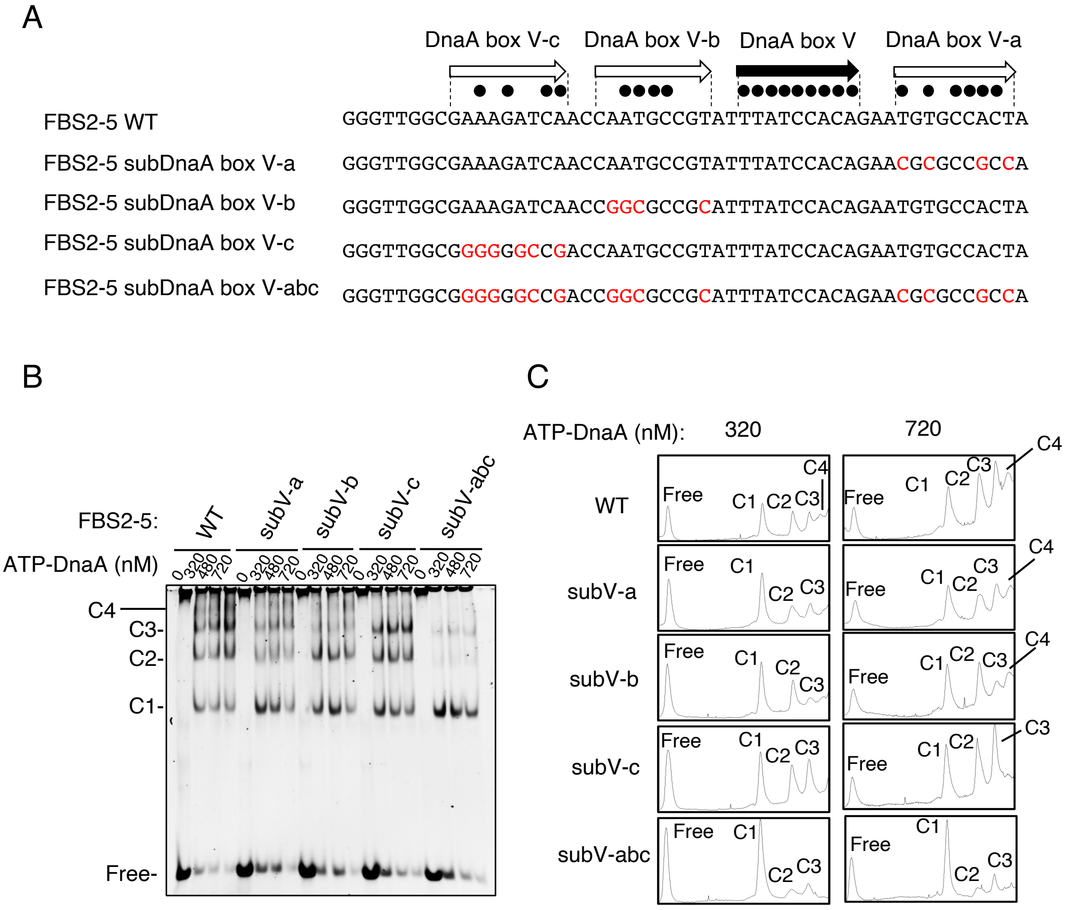

A. The sequence of low affinity DnaA box mutants. Substituted bases are displayed by red letters. **B-C**. EMSA with DnaA binding site mutants. Various concentrations of ATP-DnaA were incubated at 30°C for 5 min in buffer containing 35 nM of wild-type FBS2-5 (WT) or its mutant derivatives (subV-a, b, c, or abc), followed by EMSA using 7% PAGE. The representative gel images were shown (**B**). Free, DnaA-free-DNA; C1-C4, Complex 1-4. Each lane was analyzed densitometrically using imageJ and the profiles for 320 and 720 nM of DnaA are shown (**C**).
